# Effect of Food and a Proton-Pump Inhibitor on the Absorption of Encorafenib: An *In Vivo*–*In Vitro*–*In Silico* Approach

**DOI:** 10.1021/acs.molpharmaceut.3c00016

**Published:** 2023-04-10

**Authors:** Joseph Piscitelli, Bart Hens, Irena Tomaszewska, Lance Wollenberg, Kevin Litwiler, Mark McAllister, Micaela Reddy

**Affiliations:** † Pfizer Inc., Global Product Development, La Jolla, California 92121, United States; ‡ Pfizer Inc., Drug Product Design, Sandwich CT13 9NJ, United Kingdom; § Pfizer Inc., Early Clinical Development, Boulder, Colorado 80301, United States; ∥ Pfizer Inc., Global Product Development, Boulder, Colorado 80301, United States

**Keywords:** encorafenib, pharmacokinetics, *in vitro*, *in vivo*, *in silico*

## Abstract

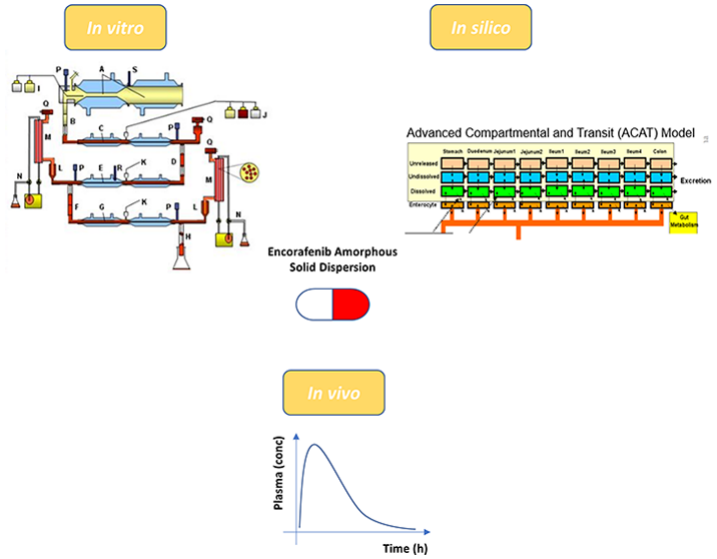

Encorafenib is a kinase inhibitor indicated for the treatment of patients with BRAF mutant melanoma and BRAF mutant metastatic colorectal cancer. To understand the effect of food and coadministration with a proton-pump inhibitor (PPI), *in vitro*, *in vivo*, and *in silico* data were generated to optimize the clinical dose, evaluate safety, and better understand the oral absorption process under these conditions. Study 1 evaluated the effect of food on the plasma pharmacokinetics, safety, and tolerability after a single oral dose of encorafenib 100 mg. Study 2 evaluated the same end points with coadministration of encorafenib and rabeprazole (PPI perpetrator). The *in vitro* gastrointestinal TIM-1 model was used to investigate the release of encorafenib and the amount available for absorption under different testing conditions (fasted, fed, and with the use of a PPI). The fasted, fed, and PPI states were predicted for the encorafenib commercial capsule in GastroPlus 9.8. In study 1, both AUC_inf_ and AUC_last_ decreased by 4% with the administration of a high-fat meal. The *C*_max_ was 36% lower than with fasted conditions. All 3 exposure parameters in study 2 (AUC_inf_, AUC_last_, and *C*_max_) had mean changes of <10% when encorafenib was coadministered with a PPI. Using the *in vitro* gastrointestinal simulator TIM-1, the model demonstrated a similar release of drug, as the bioaccessible fraction, in the 3 conditions was equal (≥80%), predicting no PPI or food effect for this drug formulation. The modeling in GastroPlus 9.8 demonstrated complete absorption of encorafenib when formulated as an amorphous solid dispersion. To obtain these results, it was crucial to integrate the amorphous solubility of the drug that shows a 20-fold higher solubility at pH 6.8 compared with crystalline solubility. The increased amorphous solubility is likely the reason no PPI effect was observed compared with fasted state conditions. The prolongation in gastric emptying in the fed state resulted in delayed plasma *T*_max_ for encorafenib. No dose adjustment is necessary when encorafenib is administered in the fed state or when coadministered with a PPI. Both the TIM-1 and physiologically based pharmacokinetic model results were consistent with the observed clinical data, suggesting that these will be valuable tools for future work.

## Introduction

Encorafenib is an oral small-molecule kinase inhibitor with potent and selective inhibitory activity against mutant BRAF kinase, a member of the RAF/MEK/ERK MAPK pathway. Mutations in the *BRAF* gene, such as BRAF V600E, in advanced stage unresectable or metastatic melanoma can result in constitutively activated BRAF kinases that may stimulate tumor cell growth. In the setting of BRAF mutant metastatic colorectal cancer (mCRC), induction of EGFR-mediated MAPK pathway activation has also been identified as a mechanism of resistance to BRAF inhibitors. Combinations of a mutant BRAF inhibitor and agents targeting EGFR have been shown to overcome this resistance mechanism in nonclinical models. Encorafenib has received marketing approvals for the treatment of patients with unresectable or metastatic BRAF mutant melanoma (450 mg orally once daily [QD] in combination with binimetinib 45 mg orally twice daily) based on the phase 3 COLUMBUS study^1,2^ and for the treatment of patients with BRAF mutant mCRC (encorafenib 300 mg orally QD in combination with cetuximab 400 mg/m^2^ initial dose followed by 250 mg/m^2^ once a week) based on the phase 3 BEACON study.^3^ Encorafenib is currently being used in combination with other targeted agents in ongoing clinical trials for the treatment of patients with other selected BRAF mutant advanced or metastatic solid tumors.

Encorafenib is an orally bioavailable drug with at least 86% of the dose being absorbed.^4^ Although classified as a Biopharmaceutics Classification System (BCS) class 2 compound, encorafenib’s formulation design resolved the issue of poor solubility, and the amorphous drug in the capsule formulation behaves as a BCS class 1 compound. The median time to reach maximum encorafenib concentration (*T*_max_) in humans occurs approximately 2 h after a single oral dose of encorafenib in the fasted state and 4 h in the fed state.^4^ The geometric mean and coefficient of variation of apparent volume of distribution is 164 L (70%).^4^ The *in vitro* protein binding of encorafenib in human plasma is 86%.^4^ The blood-to-plasma concentration ratio is 0.58. Encorafenib is primarily metabolized by CYP3A4 (83%) and to a lesser extent by CYP2C19 (16%) and CYP2D6 (1%).^4^ An increase in oral clearance with repeat dosing (apparent clearance is 14 L/h at day 1, increasing to 32 L/h at steady-state) is attributed to autoinduction of CYP3A4.^4^ Following a single radiolabeled dose of 100 mg of encorafenib, 47% of the administered dose was recovered in feces (5% unchanged) and 47% in urine (2% unchanged).^4^

The absorption of an orally administered drug can be affected by multiple intrinsic and extrinsic factors. Food effect studies are conducted to assess the effects of food on the rate and extent of absorption of a drug when it is administered shortly after a meal compared with administration under fasting conditions. Food can alter the bioavailability of a drug through various mechanisms such as delaying gastric emptying, changing the gastrointestinal (GI) pH and fluid volumes, stimulating bile flow, increasing splanchnic blood flow, changing the luminal metabolism of a drug substance, or a physical/chemical interaction with the drug in question.^5^ Because food effects mainly result from a combination of factors (including the formulation or delivery system used) that influence the solubility and dissolution of a drug, conducting a food effect relative bioavailability study is needed in most cases to understand the effect of food on the drug exposure.^6^ Additionally, the solubility and dissolution of compounds such as weak acids or weak bases can also be altered by the elevation of gastric pH by acid-reducing agents (ARAs). ARAs such as histamine H2 receptor antagonists or proton pump inhibitors (PPIs) are commonly used by cancer patients, which puts many individuals at risk for clinically relevant pH-dependent drug–drug interactions. Changes in bioavailability with the concomitant administration of an ARA or food could also affect pharmacodynamic end points, as seen with weak-base drugs and the potential for loss of efficacy due to decreased absorption of the drug in a high gastric pH setting. For these reasons, assessing the risk of drug–drug interactions is crucial to appropriately dosing encorafenib.^7^

The TIM-1 system is a dynamic, complex, multistage dissolution model developed to simulate the conditions in the stomach and the small intestine of human adults.^8^ This model is widely used to study food digestion^9−11^ and provides a measure of the bioaccessibility of nutrients and drugs from various meals and dosage forms under fed and fasting states in adults.^12−20^ The TIM system can (1) provide dynamic simulation of the gastric and small intestinal conditions relevant to various population subgroups, (2) evaluate the effect of food (and type of meal) on dosage form performance, (3) assess the impact of critical bioavailability attributes to inform dosage form design, and (4) assess dosage form performance under administration conditions that reflect clinical trial design such as simulating achlorhydric conditions seen with concomitant PPI administration. The unique setup of this model helps the formulation scientist to explore how much of the drug will be released from its drug formulation when transiting the gastric and small intestinal sections of the GI tract, considering and respecting biorelevant parameters such as fluid secretion, gastric emptying, and transit times. The physiological parameters can be adjusted to fasted, fed, and PPI conditions, as these settings are computer-controlled. Therefore, in the fed state, the gastric emptying rate can be decreased and secretions can be adjusted to reflect increased levels of bile and digestive enzymes observed after food ingestion.^21^

The application of physiologically based pharmacokinetic (PBPK) models can help the formulation scientist to further explore the impact of any formulation, physiology, or population parameter on the systemic exposure of the drug.^22−24^ Sensitivity analyses can be performed to judge which parameters play a pivotal role in the absorption process. Moreover, if any formulation changes would be made during the clinical or postapproval phase of drug product development, these changes can be explored in the modeling software to directly anticipate the impact of these changes on the systemic outcome of the drug.

To clinically characterize the effect of food on the absorption of orally administered encorafenib, study 1, a dedicated food effect study that evaluated the effect of a high-fat meal on the pharmacokinetics (PK) of encorafenib, was conducted. Additionally, study 2 evaluated the PK of encorafenib with the concomitant use of a PPI. The effect of food and PPI on single-dose encorafenib PK was evaluated in healthy participants to inform the appropriate clinical use and dose regimen of encorafenib for cancer patients. The focus of this study was to describe (1) the clinical data when encorafenib was orally dosed as an amorphous solid dispersion to healthy participants and (2) how the TIM-1 model results and mechanistic oral absorption modeling were able to describe and understand the PK and biopharmaceutical properties of encorafenib. The robust approach of *in vivo*, *in vitro*, and *in silico* approaches will assist formulation scientists to better understand drug product behavior in the human GI tract and the absorption process for these enabling formulations.

## Experimental Section

### Clinical Study Design

Study 1 was an open-label, randomized, single-dose, 2-way crossover study to determine the effect of food on the PK of encorafenib in healthy adults. Study 2 was an open-label, 2-arm, parallel-group, fixed-sequence study to investigate the effects of oral rabeprazole, a PPI, on the PK of oral encorafenib in healthy adults. Both studies were conducted at the Celerion Clinical Research Unit in Lincoln, NE. The trial protocols and informed consent documentation were reviewed and approved by the institutional review boards at the center. These studies were conducted in compliance with the ethical principles originating in or derived from the Declaration of Helsinki and in compliance with all International Council for Harmonization Good Clinical Practice Guideline.

### Eligibility of Participants

Eligible participants for both studies were male or female of non-child-bearing potential (postmenopausal or permanently infertile), aged 19–60 years, and with a body mass index ≥ 18.0 and ≤ 32.0 kg/m^2^ and a total body weight > 50 and < 113.6 kg (study 2 only). Participants must have been medically healthy with no clinically significant medical history, physical examination, ophthalmic examination, laboratory profiles, vital signs, or electrocardiograms (ECGs), as deemed by the principal investigator. Last, only continuous nonsmokers who had not used nicotine-containing products for at least 3 months prior to the first dose of study drug could be enrolled in the study.

Key exclusion criteria included (1) history or presence of hypersensitivity or idiosyncratic reaction to the study drugs or related compounds; (2) any condition possibly affecting drug absorption (e.g., malabsorption syndrome, inflammatory bowel disease, gastrectomy, gastric bleeding, gastric bypass); (3) the inability to refrain from or anticipated the use of any drug, including prescription and nonprescription medications, including vitamins, PPI, H2-antagonists or antacid preparations, herbal and dietary supplements, or grapefruit/grapefruit-containing products beginning 14 days prior to the first dose of study drug and throughout the study; and (4) a diet incompatible with the on-study diet, in the opinion of the principal investigator, within the 28 days prior to the first dose of study drug, and throughout the study.

### Study Drug Administration

In study 1, participants were randomized to 2 treatment sequences. Participants were either fasted or fed a high-fat meal on day 1 of the first period and received the alternate treatment on day 1 of the second period. Each dose was separated by a washout period of at least 7 days. A 100 mg dose of encorafenib was administered following an overnight fast (10 h prior to study drug administration and continued for at least 4 h postdose) in the fasted arm. In the food effect arm, a 100 mg dose of encorafenib was administered 30 min after the start of a high-fat breakfast. The high-fat breakfast consisted of 2 slices of buttered toast, 2 fried eggs, 2 strips of bacon, 4 oz. of hash brown potatoes, and 240 mL of whole milk. This breakfast met the criteria specified in the FDA guidance for a high-fat meal.^6^ Participants did not eat for at least 4 h following dosing. Water (except water provided with each dosing) was restricted 1 h prior to and 1 h after each study drug administration but was allowed ad libitum at all other times. Participants were housed from day −1 of each period until after the 36 h blood draw and/or study procedures on day 2. Participants returned to the clinical research unit for the 48 and 72 h blood draws.

The goal of study 2 was to assess the effect that increased gastric pH from coadministration of a PPI, such as rabeprazole, on encorafenib absorption. In the preliminary results from 15 participants who received the first dose of encorafenib (300 mg, fasted), several participants experienced adverse events (AEs) that were considered mild in severity, except 1 participant who experienced a moderate level of nausea and 1 participant who experienced a moderate level of myalgia. The AEs described were consistent with the known safety profile of encorafenib. To minimize the potential for AEs, the second encorafenib dose on day 8 was reduced to 100 mg by protocol amendment to be consistent with the dose used in a human absorption, distribution, metabolism, and excretion study (Pfizer internal data). The Study 2 protocol was further modified to add a third treatment period of single-dose encorafenib in the fasted state using 100 mg of encorafenib to provide a direct, crossover comparison of the 100 mg dose with and without rabeprazole. As a result, period 1 consisted of a single oral dose of encorafenib 300 mg; period 2 consisted of 4 oral doses of rabeprazole 20 mg QD before a fifth dose coadministered with a single oral dose of encorafenib 100 mg, and after a washout period of 28 days. Period 3 consisted of a single oral dose of encorafenib 100 mg.

Across both studies, encorafenib was administered orally (as an immediate release capsule that utilizes a hot-melt extrusion manufacturing process to produce a stable amorphous solid dispersion) with approximately 240 mL of water according to the assigned treatment schedule. Additionally, participants were instructed not to crush, split, or chew the study drug. Participants in both studies remained ambulatory or seated upright for the first 4 h following encorafenib administration, except when they were supine or semireclined for study procedures.

### Evaluation of PK and Safety

Blood samples of approximately 3 mL were collected into appropriately labeled tubes containing the anticoagulant dipotassium ethylenediamine tetra-acetic acid at each specified time point for measurement of plasma concentrations of encorafenib in both studies. Serial blood samples for PK were collected in Study 1 predose and at 0.25, 0.5, 1, 1.5, 2, 2.5, 3, 3.5, 4, 5, 6, 8, 10, 12, 24, 36, and 72 h after the day 1 dose in each period. Serial blood samples for PK were collected for Study 2 predose and at 0.5, 1, 1.5, 2, 3, 4, 6, 8, 10, 12, 24, 36, 48, and 72 h following encorafenib dosing in each period.

Plasma concentrations of encorafenib were determined using a high-performance liquid chromatography-tandem mass spectrometry method validated with respect to accuracy, precision, linearity, sensitivity, and specificity at WuXi AppTec Co, Ltd., Shanghai, China. The analytical range (from lower limit of quantification to the upper limit of quantification) for encorafenib was 1–1000 ng/mL. The PK parameters determined for encorafenib using noncompartmental analysis of the observed plasma concentration–time data are reported in Table 1. The actual sample collection times were used for the PK parameter analysis. Parameters of AUC_inf_, *t*_1/2_, CL/F, and *V_z_*/F were reported only when a well-characterized terminal phase was observed, defined as one with at least 3 data points and *r*^2^ ≥ 0.8.

**Table 1:** Noncompartmental Pharmacokinetic Parameters Calculated

parameter	definition	method of determination
AUC_0–last_	area under the concentration–time curve from time 0 to the time of the last observed/measured nonzero concentration	calculated using the linear trapezoidal with linear interpolation method
AUC_0–inf_	area under the concentration–time curve from time 0 extrapolated to infinity	AUC_0–∞_ = AUC_0–*t*_ + (*C*_last_/*k*_el_), where *C*_last_ is the last observed/measured concentration
*C*_max_	maximum observed concentration	taken directly from bioanalytical data
*T*_max_	time to reach *C*_max_	taken directly from bioanalytical data
*k*_el_	apparent first-order terminal elimination rate constant	calculated from a semilog plot of the plasma concentration versus time curve by linear least-squares regression analysis using the maximum number of points in the terminal log–linear phase (e.g., ≥3 nonzero plasma concentrations)
*t*_1/2_	apparent first-order terminal elimination half-life	calculated as 0.693/*k*_el_
CL/F	apparent total body clearance after extravascular administration	calculated as dose/(AUC_0–inf_)
*V*_*z*_/F	volume of distribution during the apparent terminal phase after extravascular administration	calculated as dose/(AUC_0–inf_ × *k*_el_)

The overall safety profile was characterized by laboratory test abnormalities, physical examination, vital signs, ECGs, and AEs. Single measurements of blood pressure and heart rate were obtained in both studies, as well as triplicate ECGs (echocardiograms), which were measured at screening, prior to, and approximately 1 h post encorafenib dosing. Triplicate ECGs were also measured at the end of the studies or upon early termination.

### Statistics

The sample size was calculated for study 1 using a power of at least 80% and an alpha error of 5%. The power was defined as the probability of having a 90% confidence interval (CI) to a ratio within the acceptance criteria of 80% to 125%. The sample size for study 2 was based on feasibility and targeted an adequate precision of at least 20% in estimation of treatment differences between binimetinib or encorafenib administered alone and in combination with rabeprazole.

To evaluate the food effect in study 1, the plasma encorafenib exposure PK parameters (AUC_last_, AUC_inf_, and *C*_max_) were ln-transformed and analyzed separately using a linear mixed-effects model. Each analysis included the calculation of treatment least-squares means (LSMs), the difference between treatment LSMs (encorafenib [fed] versus encorafenib [fasted]), and corresponding to the difference 2-sided 90% CI. These were back-transformed to obtain the geometric LSMs and ratios of LSMs (geometric mean ratios [GMRs]), and the corresponding 90% CIs of the GMRs, on the original scale. The lack of a food effect was to be concluded if the 90% CIs for the GMRs of the ln transformed, AUC_last_, AUC_inf_, and *C*_max_ of encorafenib fell between 80% and 125%.

To assess the differences between treatment (encorafenib + rabeprazole 20 mg vs encorafenib alone) for study 2, the plasma encorafenib exposure PK parameters (AUC_last_, AUC_inf_, and *C*_max_) were ln-transformed and analyzed separately using a linear mixed-effects model including treatment as a fixed effect and subject as a random effect. The differences between treatment (encorafenib 100 mg + rabeprazole versus encorafenib 100 mg alone) and corresponding 90% CIs were derived from the model. These were back-transformed to obtain the GMRs and corresponding 90% CIs on the original scale. The ratios were expressed as a percentage relative to the reference treatment.

### TIM-1 Method

To investigate the bioaccessibility under the different test conditions (i.e., in the fasted state, fed state, and with the use of a PPI), a dynamic *in vitro* GI model TIM-1 (TNO; Zeist, Netherlands) was used, which has previously been described and detailed pictorially.^25,26^ The bioaccessibility of the drug refers to the amount of drug available for drug absorption. The two filters, which are located at the end of the jejunal and ileal compartment, will capture the dissolved fraction of drug as released from the drug formulation with the help of a filtration process. The dissolved fraction of drug is the sum of drug molecules dissolved in the aqueous media (i.e., molecularly dissolved and encapsulated in the colloidal structures such as micelles). The TIM-1 model is a complex *in vitro* GI simulator that models the dynamic processes and conditions present in the lumen of the gastric and small intestinal regions of the GI tract. The TIM-1 system consists of a stomach, duodenum, jejunum, and ileum compartment. For more information about the setup of the TIM-1 model, the reader is referred to external literature.^27^ After dosing the drug product to the stomach compartment, the computer-controlled system controls the transit of the drug formulation to the intestinal compartments at a rate that reflects the *in vivo* transit conditions observed in fasted, fed, and PPI states. At the end of the jejunal and ileal compartments, a lipid filtration system is installed to separate the dissolved amount of drug from these compartments and evaluate the amount of drug available for drug absorption (i.e., the bioaccessible fraction of drug). The TIM-1 model is programmed to evaluate drug product behavior in fasted, fed, and PPI conditions.^19,28,29^ Depending on the chosen clinical condition (PPI, fed), specific experimental protocol is executed. Fasted state is simulated by implementing a predefined pH curve controlled by secretion of 1 M HCl into the gastric compartment. The gastric pH starts at pH 3 (including the coadministered glass of water) and will decrease to pH 1.7 as gastric secretion will acidify the gastric content. When the fasted PPI state is simulated, gastric content is adjusted to pH 4.5, and only water is secreted into the stomach compartment to maintain constant pH. During fed state studies, gastric pH starts at pH 6.5 and will decrease to basic fasted conditions (pH 1.7) as the food empties the stomach, and gastric secretion will be activated. Due to secretion and transit, the volumes and pH remain constant in the intestinal compartments. The gastric emptying half-life of emptying to duodenum is approximately 20 and 80 min for fasted and fed state conditions, respectively. For more information about the TIM-1 settings and the determination of bioaccessible fraction, the reader is referred to external literature.^27,28^ The bioaccessible fraction was determined by withdrawing samples from the filtrate (at the end of the jejunum and ileum compartment) and these samples were, subsequently, analyzed by HPLC. The expression of the bioaccessible fraction can be depicted in a cumulative or noncumulative order to observe how much drug will become available as a function of time. A 75 mg encorafenib capsule (containing the hot-melt-extruded encorafenib) was evaluated in the TIM-1 system. Experiments were performed in duplicate (*n* = 2) and data are presented as mean ± range.

### PBPK Modeling

Simulations were performed using the commercially available PBPK modeling platform GastroPlus 9.8 (Simulations Plus, Inc., Lancaster, CA). Input and final parameters for the model were published by Del Frari et al.^29^ All simulations were evaluated with respect to the observed geometric mean PK concentrations of encorafenib after oral administration of a 100 mg encorafenib capsule to evaluable healthy participants in studies 1 and 2. The fasted, fed, and PPI states were predicted for the encorafenib commercial capsule. All input data for the platform are shown in Table 2 and were based on physicochemical properties of the compound, formulation characteristics and absorption, distribution, metabolism, and excretion properties. Important during this model exercise was to inform the PBPK software with the amorphous solubility of the drug, which is 20-fold higher than crystalline solubility at pH 6.8. Related to dissolution, the Johnson model was selected. This model is an extension of the Nernst–Brunner dissolution model and accounts for changing particle radius during dissolution as well as for dissolution of cylindrical particles. A direct comparison with the *Z*-factor approach was made, but no major differences were observed in terms of simulated profiles. Gathered information concerning physicochemical and biopharmaceutical properties was obtained in-house. Fasted state physiology in the human GI tract was simulated by selecting the fluid volumes as observed by the magnetic resonance imaging study of Mudie et al.^30^ to address biorelevant fluid volumes in the different GI compartments. In the case of the fed state, the stomach transit time was adjusted and optimized to a value of 2.25 h for consistency with the observed PK data, and the physiology was adjusted to fed state conditions (default settings). To simulate PPI conditions, the fasted state physiology was selected (magnetic resonance imaging fluid volumes). However, gastric pH was set to a constant value of pH 7 during the simulation instead of the normal gastric pH of 1.3, as used in the fasted state default settings. The gastric pH after dosing a PPI for 3 consecutive days was shown to be approximately pH 7 in healthy individuals. However, the same results were observed even when the gastric pH was adjusted to a value of 4 or 5 (data not shown).^31^ Distribution and clearance parameters were adjusted to reflect a 2-compartmental approach based on the average PK profile of the 100 mg dose as administered in the fasted state to healthy participants (average weight, 83 kg). Although encorafenib is a CYP3A autoinducer (i.e., its exposures at steady state were lower than exposures for the first dose in studies conducted in cancer patients), the PBPK model did not include autoinduction because simulations were only needed for a single dose of encorafenib. Finally, a parameter sensitivity analysis was performed where the stomach transit time varied from 0.25 to 3 h, and the impact on fraction absorbed, plasma *C*_max_, *T*_max_, and AUC was explored.

**Table 2:** PBPK Model Parameters

parameter	value	source
log*P*	2.59	experimental data
log*D*	1.8 at pH 6.8	experimental data
diffusion coefficient	0.55 × 10^–5^ cm/s^2^	predicted (ADMET Predictor)
p*K*_a_	base: 4.53; acid: 6.97	experimental data
reference solubility (amorphous)	0.2 mg/mL at pH 6.8	experimental data
reference solubility (crystalline)	0.01 mg/mL at pH 6.8	experimental data
solubility factor	1000	estimated using *in vitro* data
bile salt solubilization ratio	127000	estimated using *in vitro* data
human effective permeability (*P*_eff_)	3.01 × 10^–4^ cm/s	estimated using *in vivo* data
particle radius	5 μm	experimental data
precipitate radius	1 μm	GastroPlus default value
drug particle density	1.2 g/mL	GastroPlus default value
mean precipitation time	90000 s	estimated
clearance	16.08 L/h^31^	estimated using PKPlus module
K10	0.197 h^–1^^31^	estimated using PKPlus module
K12	0.173 h^–1^^31^	estimated using PKPlus module
K21	0.016 h^–1^^31^	estimated using PKPlus module
liver CYP2C19 *K*_m_ and *V*_max_	7.51 mg/L and 0.00504 mg/s^31^	fitted using *in vivo* data
gut CYP2C19 *K*_m_ and *V*_max_	7.51 mg/L and 0.00504 mg/s^31^	fitted using *in vivo* data
liver CYP3A4 *K*_m_ and *V*_max_	213.9 mg/L and 0.0945 mg/s^31^	fitted using *in vivo* data
gut CYP3A4 *K*_m_ and *V*_max_	213.9 mg/L and 0.0945 mg/s^31^	fitted using *in vivo* data

## Results

### Participant Disposition and Baseline Characteristics

Thirty-one participants completed study 1: 19 participants completed the reference arm (fasted state) first, and 21 participants completed the treatment arm (fed state) first. Of the 40 participants who entered study 1, 9 discontinued early, including 6 who discontinued due to AEs. Study 2 included a total of 15 participants: 10 participants completed the study per protocol, and 1 discontinued due to an AE. Participant demographics across both studies at baseline are summarized in Table 3.

**Table 3:** Demographic Summary^a^

trait	category/statistic	study 1	study 2
sex, *n* (%)	female	9 (23)	4 (27)
	male	31 (78)	11 (73)
race, *n* (%)	Black or African American	2 (5)	7 (47)
	White	37 (93)	8 (53)
	White, Black, or African American	1 (3)	N/A
ethnicity, *n* (%)	Hispanic or Latino	4 (10)	1 (7)
	Not Hispanic or Latino	36 (90)	14 (93)
age, years	*n*	40	15
	mean	43.1	38.1
	SD	11.71	12.95
	median	46.0	36.0
	minimum	22	19
	maximum	60	56
weight, kg	*n*	40	15
	mean	83.80	78.21
	SD	14.094	11.664
	median	84.20	78.70
	minimum	59.5	58.5
	maximum	109.3	95.4
height, cm	*n*	40	15
	mean	176.2	172.3
	SD	7.89	10.07
	median	176.0	173.0
	minimum	161	147
	maximum	192	190
BMI, kg/m^2^	*n*	40	15
	mean	26.882	26.264
	SD	3.5390	2.6521
	median	26.775	27.150
	minimum	20.27	20.78
	maximum	31.76	31.34

a Abbreviations: BMI = body mass index; SD = standard deviation.

### PK Results

**Figure 1:**
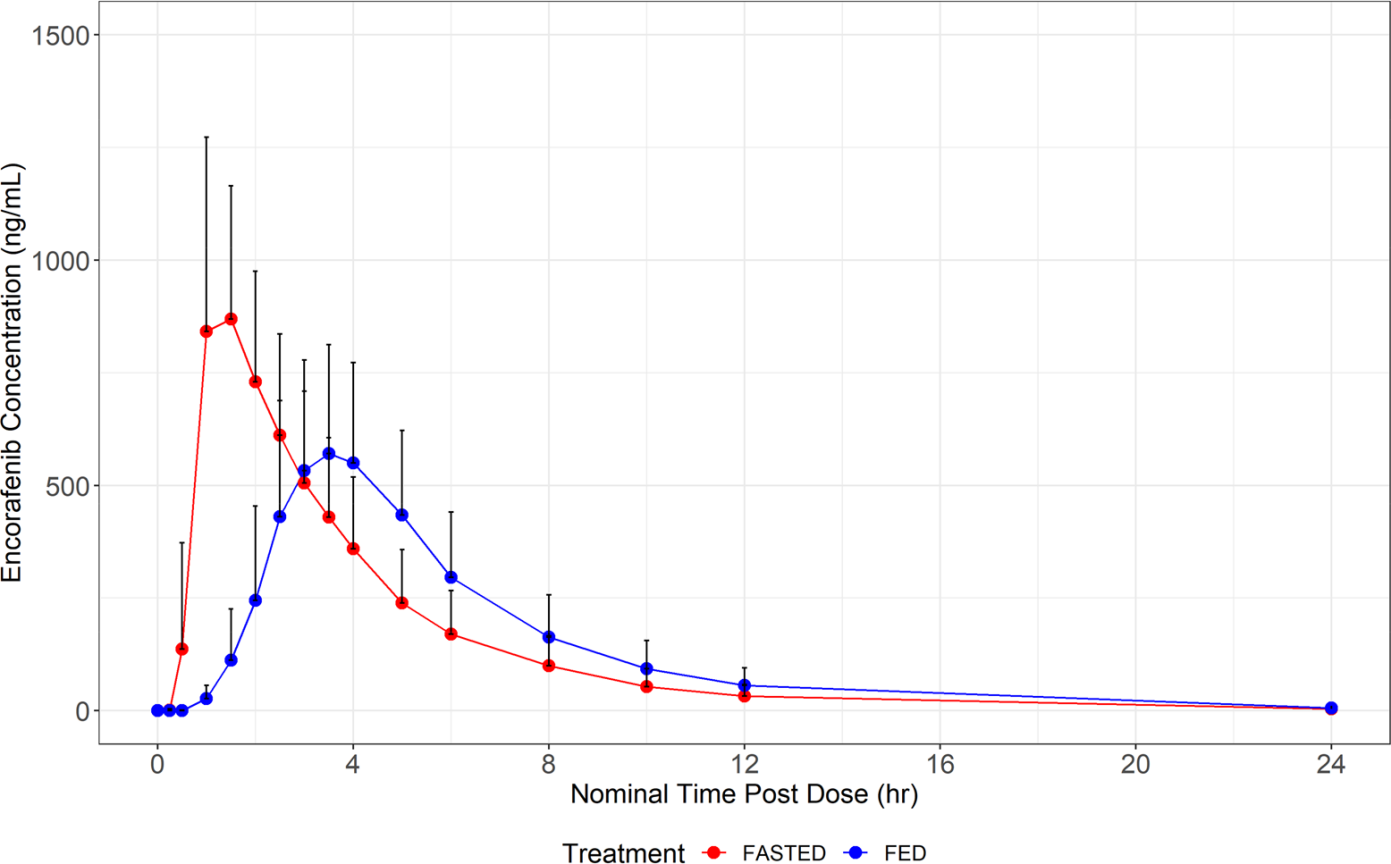
Mean encorafenib concentrations (+ standard deviation) for study 1. Observed mean encorafenib concentrations over time after taking encorafenib 100 mg in the fasted (red) or fed (blue) states. The solid dots represent the mean encorafenib concentration at the time point specified. The black lines represent the upper standard deviation of the encorafenib concentration at each time point

**Figure 2:**
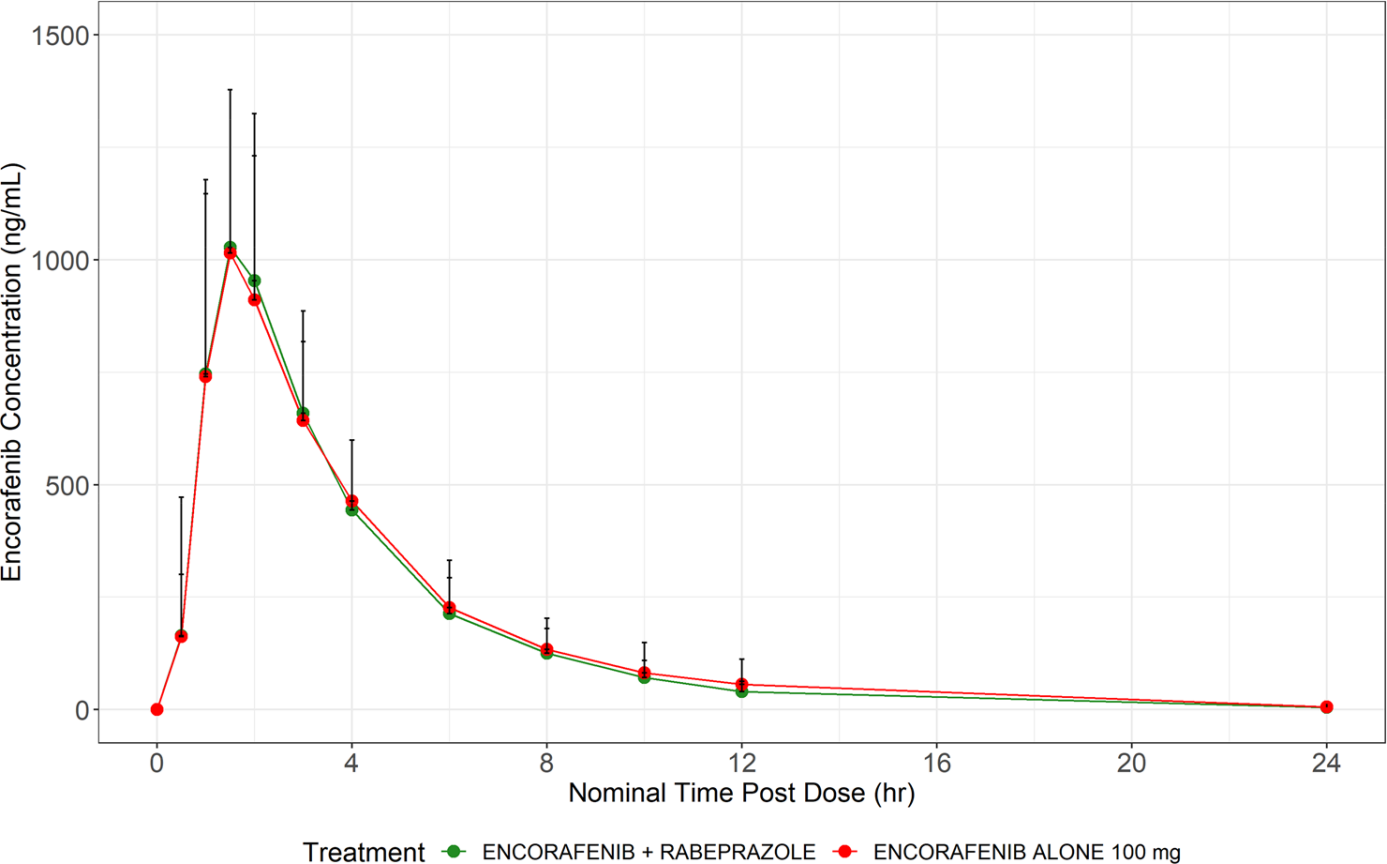
Mean concentrations for encorafenib (+ standard deviation) for study 2. Observed mean encorafenib concentrations over time after taking encorafenib 100 mg alone (red) or in combination with rabeprazole (green). The solid dots represent the mean encorafenib concentration at the time point specified. The black lines represent the upper standard deviation of the encorafenib concentration at each time point.

The observed plasma concentration versus time profiles and PK parameter estimates for encorafenib in participants for studies 1 and 2 are presented in Figures 1 and 2 and Table 4, respectively.

**Table 4:** Summary of Plasma Encorafenib Pharmacokinetic Parameter Values Following Single Oral Doses of Encorafenib 100 mg in Studies 1 and 2^a^

	parameter summary statistics by treatment					
	study 1 (food effect)			study 2 (PPI)		
parameter	encorafenib (fed) GM (GCV%)	encorafenib (fasted) GM (GCV%)	geometric mean ratio (90% CI)	encorafenib alone GM (GCV%)	encorafenib + rabeprazole GM (GCV%)	geometric mean ratio (90% CI)
*N*, *n*^b^	31, 31	31, 30		10, 10	11, 11	
AUC_inf_, ng·h/mL	3034 (38.4)	3121 (42.3)	95.91 (91.62–100.39)	4137 (33.4)	4014 (35.7)	96.58 (76.62–121.75)
AUC_last_, ng·h/mL	3018 (38.6)	3129 (41.9)	95.81 (91.66–100.14)	4119 (33.4)	3991 (35.9)	96.47 (76.40–121.81)
*C*_max_, ng/mL	621.0 (45.2)	961.5 (32.9)	63.97 (57.73–70.90)	1041 (36.9)	974.0 (70.0)	94.19 (59.32–149.56)
CL/F, L/h	35.13 ± 12.535	34.64 ± 14.209		25.33 ± 8.0601	26.31 ± 9.1417	
*T*_max_, h	3.499 (2.00, 5.00)	1.495 (0.997, 3.49)		1.503 (1.01, 2.00)	2.000 (1.50, 3.01)	
*V*_*z*_/F, L	261.7 ± 162.61	226.1 ± 207.19		246.8 ± 208.24	332.3 ± 336.32	
*t*_1/2_, h	5.612 ± 3.6862	4.790 ± 3.7930		7.529 ± 7.5768	9.175 ± 9.7473	

a Geometric mean ratio (GMR) = 100 × (test/reference). Geometric mean (% geometric CV) for all except median (range) for *T*_max_ and arithmetic mean (±SD) for *t*_1/2_. %CV = percent coefficient of variation; AUC_inf_ = area under the plasma concentration–time profile from time zero extrapolated to infinite time; AUC_last_ = area under the plasma concentration–time profile from time zero to the time of the last quantifiable concentration (C_last_); CL/*F* = apparent clearance after oral dose; *C*_max_ = maximum plasma concentration; GCV% = coefficient of variation; SD = standard deviation; *t*_1/2_ = terminal elimination half-life; *T*_max_ = time for *C*_max_; *V_z_*/F = apparent volume of distribution after oral dose.

b *N* = number of participants included in the parameter summary; *n* = number of participants with reportable AUC_inf_, *t*_1/2_, *V_z_*/F, and CL/F.

Following a single oral 100 mg administration of encorafenib in the fed state, the extent of exposure (AUC) to encorafenib was similar to that measured following a single oral 100 mg administration of encorafenib in the fasted state. Both AUC_inf_ and AUC_last_ decreased by 4%, indicating no statistically or clinically significant change in either parameter. The rate of absorption of encorafenib was slower under fed conditions than under fasted conditions, with a median *T*_max_ occurring 2 h later (1.5 vs 3.5 h) under fed conditions. The *C*_max_ for encorafenib in the fed state was 36% lower (90% CI: 29%–43%) compared with fasted conditions. Based on these data, encorafenib can be taken with or without food at the approved dose without restriction.

The exposure to encorafenib following coadministration of encorafenib 100 mg with multiple oral once-daily doses of rabeprazole 20 mg was similar to that measured following encorafenib 100 mg alone. All 3 exposure parameters (AUC_inf_, AUC_last_, and *C*_max_) for the combination had mean changes of <10%. The rate of absorption was similar following the coadministration of encorafenib 100 mg with multiple oral once-daily doses of rabeprazole 20 mg compared with that following encorafenib 100 mg alone, with comparable median *T*_max_ values. Median *T*_max_ of 2.00 h was observed following the coadministration of encorafenib 100 mg with multiple oral once-daily doses of rabeprazole 20 mg and 1.5 h following the administration of encorafenib 100 mg alone. Statistical comparisons of PK parameters for encorafenib 100 mg coadministered with rabeprazole versus encorafenib 300 mg alone are shown in Table S1.

### Safety

Encorafenib administered as a single oral dose of 100 mg was tolerated among healthy participants. However, the 300 mg dose appeared to be tolerated with more difficulty in these healthy participants than the 100 mg dose.

In study 1, AEs were reported by 38 participants (95%; all mild in severity), with 27 participants (79%) following encorafenib in the fasted state and 34 participants (92%) following encorafenib in the fed state. The most common AE was flushing, reported by 33 participants (83%), followed by headache (29 participants [73%]). Nine participants discontinued during the study; of these, 2 discontinued due to an AE related to encorafenib (headache, facial flushing, paraesthesia, generalized pruritus, and oral herpes).

In study 2, AEs were reported by 15 of 15 participants. The majority of AEs were grade 1/mild severity, and 3 AEs were grade 2/moderate severity (headache, nausea, and myalgia). The most common AEs were headache (93%), flushing (80%), and feeling hot (53%). 106 AEs were reported by the 15 participants (100%) who received encorafenib 300 mg alone. 23 AEs were reported by 9 of 11 participants (82%) who received encorafenib 100 mg + rabeprazole. 24 AEs were reported by 9 of 10 participants (90%) who received encorafenib 100 mg alone. Five participants discontinued the study early: The principal investigator discontinued 1 participant from the encorafenib arm due to vomiting, and 4 others discontinued by choice.

No clinically significant changes in vital signs, laboratory values, or ECG measurements were observed during either of studies.

### TIM-1 Results

**Figure 3:**
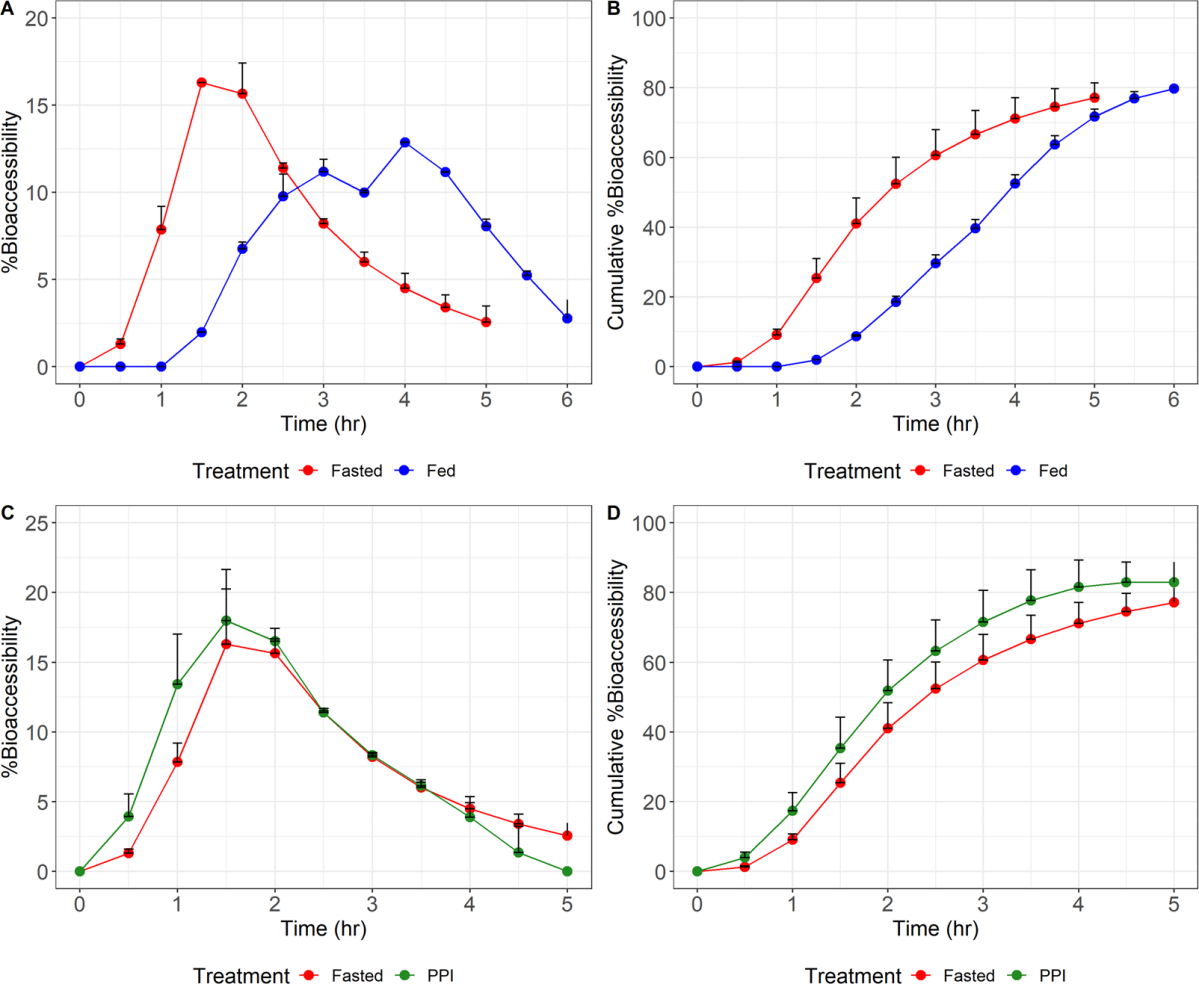
Mean % bioaccessibility or cumulative % bioaccessibility (+standard deviation) for encorafenib 75 mg TIM-1 experiments. Observed mean TIM-1 % bioaccessibility over time with a 75 mg encorafenib tablet in the fasted state (red) compared to the fed state (blue) or under the state of PPI coadministration (green). The solid dots represent the mean TIM-1 % bioaccessibility at the time point specified. The black lines represent the upper standard deviation of the TIM-1 % bioaccessibility at each time point.

Bioaccessibility data obtained from the TIM-1 filtration system are presented in Figure 3 and Table 5. Maximum bioaccessibility for the fasted state was 16%, which was comparable to the bioaccessibility determined using conditions simulating PPI coadministration (18%). Both maximum bioaccessibility estimates occurred at the same time point for the fasted and PPI predictions, which matches the clinical data. Maximum bioaccessibility for the TIM-1 simulated fed condition had a lower value of 13% and occurred at the 4-h time point. This slight decrease and delay in maximum bioaccessibility match the trend seen with the clinical study. The total bioaccessibility for the fasted, PPI, and fed states was 77%, 83%, and 80%, respectively. The experimental error between each of the TIM-1 runs was small, with the standard deviations in each state being <10%. Additionally, the differences in mean total bioaccessibility between the 3 experimental states were <10%, suggesting that changes in clinical AUC are unlikely between the fasted, fed, and PPI states.^25^

**Table 5:** Summary of Encorafenib 75 mg Capsule TIM-1 Experiments

bioaccessibility data^a^	fasted	PPI	fed
maximum bioaccessibility, %	16 ± 4	18 ± 4	13 ± 0
total bioaccessibility (cumulative), %	77 ± 4	83 ± 6	80 ± 1
time of maximum bioaccessibility, h	1.5	1.5	4

a Arithmetic mean and (± standard deviation) for all parameters except for time of maximum bioaccessibility for which only the time point is presented. PPI = proton pump inhibitor. The bioaccessibility of the drug refers to the amount of drug available for drug absorption.

These experiments show that even though there is minor change in maximum bioaccessibility, the total bioaccessibility is still similar among the 3 conditions, indicating that exposure in humans will not be significantly different. Based on the clinical data as discussed in the previous paragraphs, it can be concluded that the TIM-1 model was able to reflect the *in vivo* performance of the drug product for all 3 test conditions (i.e., fasted, fed, and PPI states).

### PBPK Modeling Results

**Figure 4:**
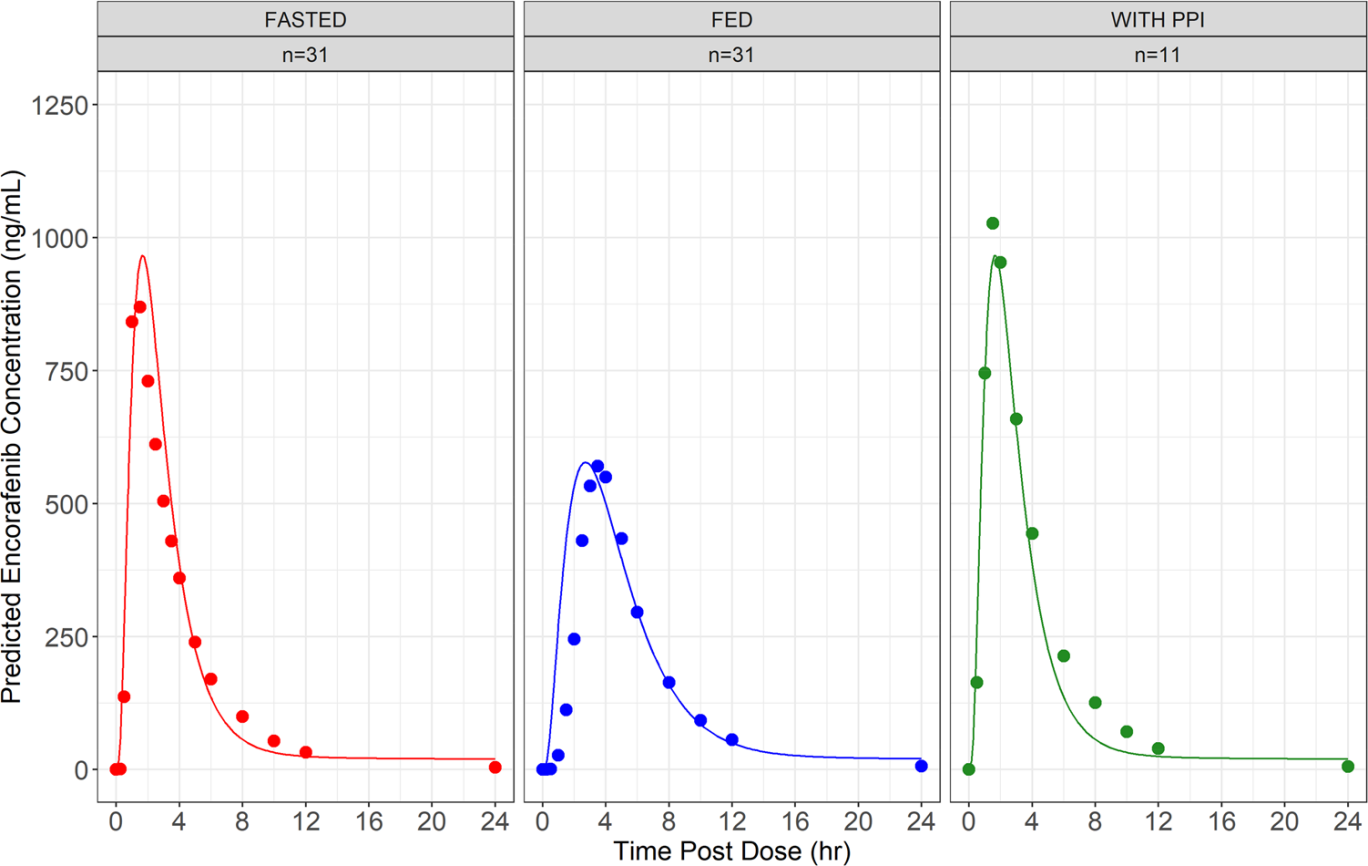
PBPK plasma predictions. Observed and simulated encorafenib exposures as a function of time following administration of a single 100 mg dose of encorafenib in fasted, fed, and fasted with coadministration conditions. Simulations are depicted by the solid lines, whereas the mean encorafenib concentration observed at each time point are depicted as dots. *n* = number of participants used to calculate the mean encorafenib concentration at each time point.

Figure 4 depicts the observed and predicted systemic concentrations of encorafenib after oral administration of a 100 mg immediate-release capsule fasted, fed and in combination with a PPI. The results of the PBPK model adequately match the observed results. The fluid volume model used for the fasting and concomitant use of PPI simulations was the “Human–Dyn Vol 100% Mudie–Fasted” model. The “Human–Physiological–Fed” model was used for the encorafenib simulations in the fed conditions. The stomach transit time for the PBPK fed state simulations was 2.25 h. This transit time was significantly different than both the fasted state simulation (stomach pH 1.3) and concomitant use of PPI simulation (stomach pH 7), which both had a stomach transit time of 0.35 h. As the formulation design resolved the issue of poor solubility, the amorphous form of the compound behaves as a BCS class 1 compound rather than a BCS class 2 compound. In the case of BCS class 1 compounds, it is generally known that the rate-limiting step for absorption is the gastric emptying rate. Therefore, a sensitivity analysis (Figure S2) was performed to address the impact of gastric emptying on the systemic disposition parameters (i.e., fraction absorbed, plasma *C*_max_, *T*_max_, and AUC). Results indicated no impact on fraction absorbed or plasma AUC as the entire dose will be absorbed. However, a decrease in plasma *C*_max_ was predicted when stomach transit time would increase. In other words, a delay in gastric emptying will delay the absorption process, therefore resulting in a decreased plasma *C*_max_. The prolonged gastric emptying will eventually also result in a delayed plasma *T*_max_. These results point out the BCS class 1 behavior of encorafenib when formulated as an amorphous solid dispersion. Similar results were observed for paracetamol when used as a tracer for gastric emptying in a patient population.^32^ For all 3 test conditions, the predicted profiles matched adequately with the observed profiles showing a complete absorption process for the 100 mg dose for all conditions. The crucial factor in the simulation is the addition of the amorphous solubility that is 20-fold higher than the crystalline solubility at pH 6.8, circumventing the low aqueous solubility in this region and, therefore, promoting the absorption process.

## Discussion

### Formulation

Encorafenib is formulated as an immediate-release capsule that utilizes a hot-melt extrusion manufacturing process to produce a stable amorphous solid dispersion to increase the aqueous solubility and, therefore, the bioavailability of encorafenib. The equilibrium solubility data of both encorafenib and hot melt extrudate at the maximum proposed dose of 450 mg is above the BCS high solubility boundary, which is defined as 1.8 mg/mL (450 mg divided by 250 mL of aqueous buffer) at pH 1 and simulated gastric fluid and below the boundary of pH 4–7.5. While both the drug substance and hot melt extrudate are below the BCS high solubility limit of pH 4–7.5, the melt extrudate is approximately an order of magnitude more soluble in this pH range, which is the typical pH range for the small intestine (pH 4.5–6.8), and this accounts for the increase in the bioavailability of encorafenib when administered as an amorphous solid dispersion.

Although the aqueous solubility of encorafenib is high at normal gastric pH, it is not sufficiently high across the full range of physiologically relevant pH values of the GI tract for encorafenib to be characterized as a highly soluble compound according to the BCS classification system. Because encorafenib demonstrates high apparent permeability, its aqueous solubility in media with higher pH results in encorafenib being designated as a BCS class 2 drug. In contrast, however, the amorphous drug product exhibits characteristics of a class 1 high-solubility, high-permeability compound *in vivo*, including lack of food effect, lack of effect due to increased gastric pH with PPI, short *T*_max_ (in fasted state), and approximate dose proportionality based on single doses up to 700 mg QD. Thus, while in vitro crystalline solubility is relatively low (0.01 mg/mL at pH 6.8), *in vivo* data suggest that solubility is not limiting with respect to performance of the drug product due to fact that the amorphous solubility was measured and resulted in a 20-fold higher value (0.2 mg/mL at pH 6.8).

### Clinical Study Observations

Studies 1 and 2 were designed with the intent to determine if there were significant differences in encorafenib PK that could clinically affect exposure. In the presence of food or a PPI, there was no clinical difference in the AUC_inf_, AUC_last_, or *C*_max_ for either study. The only PK parameter across both studies that had a statistically significant change from the fasted state was when encorafenib was administered with a high-fat meal. In this case, the *C*_max_ in the fed state was 36% lower than in the fasted state. Because there was no change in AUC between fed and fasted states, there is no clinically relevant difference in exposure.

Encorafenib exposures from the clinical studies are generally consistent when considering the PK variability [e.g., the geometric mean (geo CV%) AUC_inf_ for the fasted state in the food effect study was 3121 (42.3%, n = 30) and for the encorafenib alone group administered the same dose in the fasted state in the PPI study was 4137 (33.4%, n = 11)] however the parallel nature of statistical comparison across studies is challenging. Factors such as age, sex, body weight (body weights were 5 kg heavier on in the study with lower exposures noted), mild hepatic impairment, and more may have a minor effect on PK, but are not thought to have a clinically meaningful effect on encorafenib PK.

No dosing adjustments are necessary for coadministration of food or a PPI with encorafenib in either the encorafenib package insert or summary of product characteristics. These dosing recommendations are very important as they allow for patients to have more flexibility with encorafenib dosing (e.g., schedule their dosing around meals). Additionally, many cancer patients taking ARAs should not be concerned about their choice of ARA affecting the PK of encorafenib to a clinically significant extent.

### TIM-1 and PBPK Results Compared With Clinical Data: The Value of *In Vitro* and *In Silico* Tools

When evaluating a single 75-mg encorafenib capsule in the TIM-1 system, trends in maximum and total bioaccessibility were similar to those seen with AUC and *C*_max_ across the range of different clinical administration conditions studied. This dose differs from that used in the 2 clinical studies; however, encorafenib is approximately dose proportional in the dose range of 50 to 700 mg QD after a single dose,^33^ allowing for the results to be applied to the clinically approved doses for both BRAF mutant melanoma (450 mg orally QD) and BRAF mutant mCRC (300 mg orally QD).

Total bioaccessibility is a measure of how much drug is released from the formulation and available for absorption in the TIM-1 system cumulatively over time. Across the fasted, fed, and PPI states in the system, total bioaccessibility reflected the clinical data, showing similar total absorption for all 3 environments. Maximum bioaccessibility is a measure of the maximum percentage of encorafenib available for absorption at a specific time and also similar compared with the clinical data for all 3 conditions. Additionally, the time of maximum bioacccessibility resembled the clinical data. The TIM-1 system accurately mimicked the decrease in maximum bioaccessibility and later time of maximum bioaccessibility observed in the clinical data for participants who took encorafenib with food.

The TIM-1 system proved to be a potentially useful tool in reproducing the qualitative trends observed across the 3 dosing conditions. Due to the multistage and biorelevant setup of the TIM-1 model, we were able to evaluate the drug release across the different GI compartments and noticed an almost full release in the upper GI tract for all test conditions. During the stage of drug product development, this tool can be extremely useful to provide guidance in the selection of a lead formulation for the clinical stage. Multiple candidate formulations can be tested in a (relative) short period of time at a fraction of the cost of a clinical study. The formulation with the highest bioaccessible fraction tested in the TIM-1 system can be pushed forward to the clinical stage, as this candidate may result in an optimized *in vivo* performance (in terms of release and bioavailability of the drug). Due to different relevant barriers that are included in the TIM-1 system (e.g., bicarbonate buffer, gastric emptying times, intestinal transit times, secretions, etc.), the bioaccessible fraction can be used as a good indicator to explore the *in vivo* performance of the drug, as also shown for other compounds in the literature.^18,19^ Accurately predicting how a compound will release in the clinical setting can assist reformulation efforts, as well as in addressing mechanistic questions related to absorption. It should be noted that the TIM-1 model lacks the mucus layer as present at the apical side of the enterocytes, as well as metabolic enzymatic activity (CYP enzymes).

As shown by the results of the PBPK model, it should be noted that dissolution is not the rate-limiting step in the absorption nor the permeability. The design of the formulation (i.e., amorphous solid dispersion) overcomes the issues of low solubility and poor dissolution. Moreover, the presence of the polymer polyvinylpyrrolidone vinyl alcohol results in a sustainable degree of supersaturation in the luminal environment of the human GI tract. Precipitation time was increased from GastroPlus default value of 900–90000 s to decrease precipitation potential since systemic data are consistent with drug staying in solution. In a two-stage transfer study, a minimal amount of precipitated drug was observed after dumping the gastric content in intestinal media (data not shown). However, this method may overestimate precipitation due to (1) the dumping process and (2) the lack of an absorptive environment present in the setup. With respect to the achlorhydric conditions, the elevated gastric pH did not show any negative impact on the clinical PK parameters for *C*_max_ and AUC compared with fasted state conditions as the dissolution of the drug results in a rapid and full release of the drug, regardless of pH conditions. In the case of fed state conditions, the gastric emptying rate is presumably the rate-limiting step in the absorption process where the ingested calories result in a delayed gastric emptying process (as shown by a sensitivity analysis in the modeling workspace), therefore reducing the plasma *C*_max_ and increasing *T*_max_ compared with the fasted state conditions. The PBPK model provided insight into the mechanisms underlying the observed PK in the fed state compared with the fasted state.

## Conclusion

In summary, it was important to study the effects of both food and ARAs on the absorption of encorafenib to provide more flexibility for patients. Two separate studies were subsequently conducted to assess the effect of a high-fat meal on the absorption of encorafenib, as well as the effect of a PPI on the PK of encorafenib. Compared with participants in the fasted state, there was no clinically meaningful change in encorafenib exposure in participants in the fed state or those coadministering an ARA. Based on the findings of this research, encorafenib can be taken in both the fed or fasted state at the clinically recommended doses, and no dosing adjustment is needed when encorafenib is coadministered with an ARA. The TIM-1 and PBPK model results were consistent with the observed encorafenib clinical data. This consistency suggests that these tools will be valuable for future work, including supporting the development for new encorafenib dose strengths and formulations.

## Supporting Information Available

Summary of statistical comparisons of plasma encorafenib pharmacokinetic parameters for encorafenib 100 mg coadministered with rabeprazole versus encorafenib 300 mg alone (dose normalized to 100 mg), stomach transit time sensitivity analysis (PDF)
