# Supplementary material for: Effect of Food and a Proton-Pump Inhibitor on the Absorption of Encorafenib: An In Vivo–In Vitro–In Silico Approach
Source: Mol Pharm. 2023 Apr 10;20(5):2589–99. doi: 10.1021/acs.molpharmaceut.3c00016 (PMC10155203; doi:10.1021/acs.molpharmaceut.3c00016)

Effect of Food and a Proton-Pump Inhibitor on the Absorption of Encorafenib: An *In Vivo-In Vitro-In Silico* Approach

Joseph Piscitelli[*1], Bart Hens[2], Irena Tomaszewska[2], Lance Wollenberg[3], Kevin Litwiler[4], Mark McAllister[2], Micaela Reddy[3]

[1]Pfizer Inc, Global Product Development, La Jolla, CA

[2]Pfizer Inc, Drug Product Design, Sandwich, UK

[3]Pfizer Inc, Early Clinical Development, Boulder, CO

[4]Pfizer Inc, Global Product Development, Boulder, CO (affiliation at the time the study was conducted)

***Corresponding Author:** Joseph Piscitelli, Clinical Pharmacology, Pfizer Inc, 10555 Science Center Drive, La Jolla, CA 92121; Phone: +1-919-428-4681; Email: Joseph.Piscitelli@pfizer.com

Keywords: encorafenib; pharmacokinetics; *in vitro*; *in vivo*; *in silico*

Supplemental Tables and Figures

Table S1. Summary of Statistical Comparisons of Plasma Encorafenib Pharmacokinetic Parameters for Encorafenib 100 mg Coadministered With Rabeprazole Versus Encorafenib 300 mg Alone (Dose Normalized to 100 mg)

Parameter	Parameter Summary Statistics[a] by Treatment		
	Study 2 (PPI)		
	Encorafenib 300 mg Alone GM (GCV%)	Encorafenib 100 mg + Rabeprazole GM (GCV%)	Dose Normalized Geometric Mean Ratio (90% CI)
N, n[b]	15, 15	11, 11	–
AUC_{inf}, ng.h/mL	14800 (18.3)	4014 (35.7)	82.21 (72.40-93.34)
AUC_{last}, ng.h/mL	14740 (18.2)	3991 (35.9)	82.11 (72.22-93.36)
C_{max}, ng/mL	3895 (23.9)	974.0 (70.0)	75.17 (56.61-99.82)
CL/F, L/h	20.59 ± 3.9043	26.31 ± 9.1417	–
T_{max}, h	1.501 (1.00, 2.01)	2.000 (1.50, 3.01)	–
Vz/F, L	469.8 ± 339.16	332.3 ± 336.32	–
$t_{½}$, h	15.88 ± 11.626	9.175 ± 9.7473	–

Geometric mean ratio (GMR)=100 × (test/reference).
[a] Geometric mean (% geometric CV) for all except median (range) for T_{max} and arithmetic mean (± SD) for $t_{½}$.
[b] N=number of participants included in the parameter summary; n=number of participants with reportable AUC_{inf}, t½, Vz/F, and CL/F.
Abbreviations: %CV=percent coefficient of variation; AUC_{last}=area under the plasma concentration–time profile from time zero to the time of the last quantifiable concentration (C_{last}); AUC_{inf}=area under the plasma concentration–time profile from time zero extrapolated to infinite time; CL/F=apparent clearance after oral dose; C_{max}=maximum plasma concentration; SD=standard deviation; t1/2=terminal elimination half-life; T_{max}=time for C_{max}; Vz/F=apparent volume of distribution after oral dose.

Figure S2. Stomach Transit Time Sensitivity Analysis: The outcome of a parameter sensitivity analysis in which stomach transit time varied from 0.25 to 3 hours. The impact of stomach transit time was explored on fraction absorbed, plasma Cmax, Tmax, and AUC for a 100 mg dose of encorafenib under fasted state conditions.

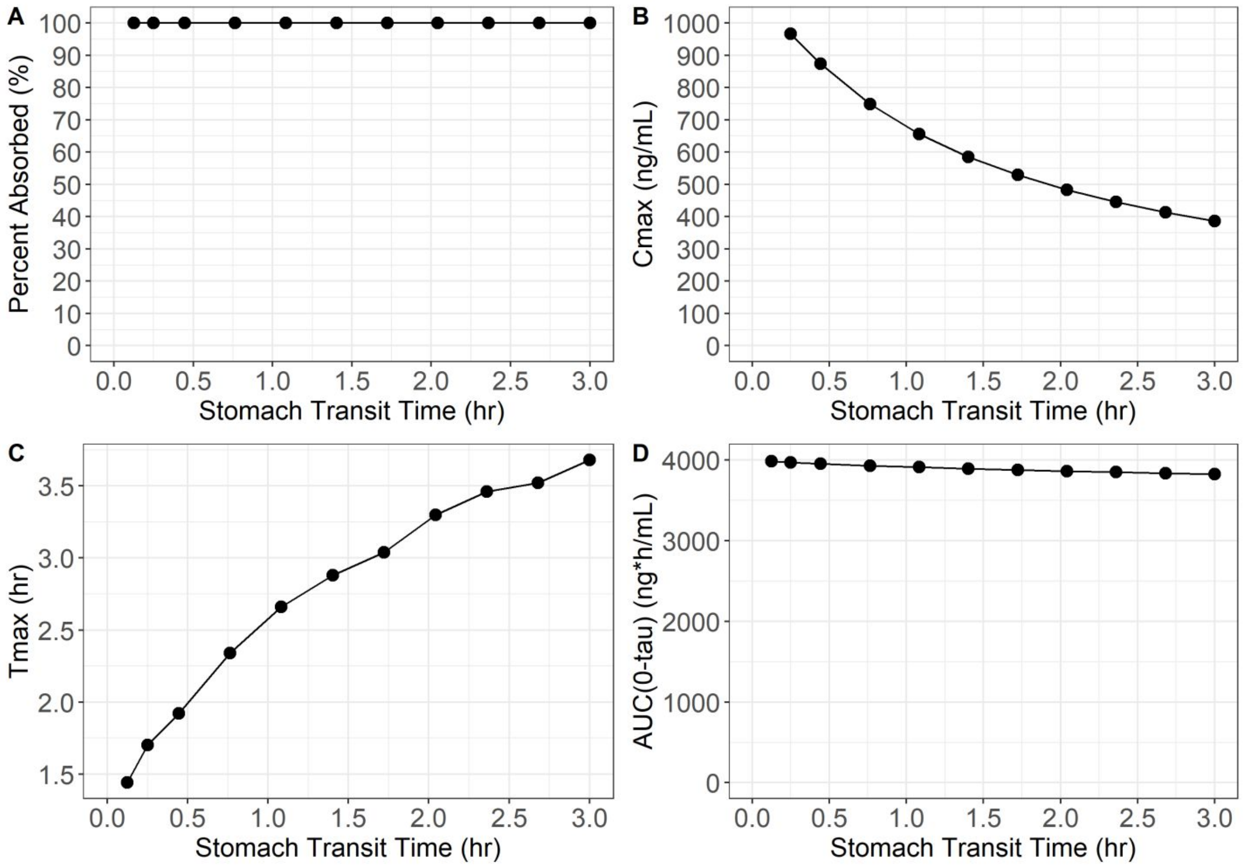

Supplement: Supplementary file 1 — mp3c00016_si_001.pdf [file mp3c00016_si_001.pdf]
